# Productive or Transforming? How Epithelial Compartmentalization of HPV DNA and E6/E7 Transcripts Shapes Cervical Screening Assay Performance

**DOI:** 10.1002/jmv.71196

**Published:** 2026-10-27

**Authors:** Konstanze Schichl, John Doorbar

**Affiliations:** ^1^ Department of Pathology University of Cambridge Cambridge UK

## Abstract

The human papillomavirus (HPV) life cycle is linked to epithelial cell differentiation, with viral genome amplification, E6/E7 transcription, and virion assembly spatially compartmentalized within the cervical epithelium. This organization is disrupted in transforming lesions. These fundamental observations determine what ‘bulk’ nucleic acid amplification tests (NAATs) of cervical exfoliates allow us to establish. DNA‐based assays sensitively report high‐risk HPV genome presence, which includes transient infections and virus particles. RNA‐based assays target the E6/E7 transcripts in transcriptionally active disease, which accounts for their slightly higher clinical specificity for high‐grade squamous intraepithelial lesions. No single assay defines both viral presence and disease significance, however. Cytology, p16/Ki‐67 dual stain, extended genotyping and host‐cell methylation interrogate complementary axes of risk with strong biological rationale. The loss of spatial information that limits bulk NAAT approaches matters most in mixed‐grade lesions and immunocompromised populations. Emerging spatial methods, AI‐assisted cytology and cervical cell‐lifts offer recovery of biological resolution.

## 1 Introduction

Persistent infection with high‐risk human papillomaviruses (hrHPV) is a necessary cause of more than 95% of cervical cancers [1]. In recent years, the replacement of cervical cytology‐based screening with primary hrHPV testing has improved screening sensitivity, because it allows the identification of an ‘at risk’ group, especially when used to screen women over 30 years of age who may be persistently HPV positive when sequential tests are carried out [2]. In younger women, where transient (incident) infections predominate, the implementation of HPV nucleic acid tests is less obviously useful, with cytology‐based screening remaining an important approach for the identification of high‐grade squamous intraepithelial lesions (HSIL; i.e. clinically relevant disease), and with visual inspection with acetic acid (VIA) being used as an alternative to this in low resource settings [3]. Although the HSILs that cytology and VIA detect are generally regarded as potential cancer precursors, in reality only a subset of HSIL are at risk of progression [4]. Indeed, the division of HSIL into CIN2 (cervical intraepithelial neoplasia 2) and CIN3 categories reflects the latter's more immediate cancer risk. CIN2 and CIN3 differ substantially in disease outcomes when untreated. In a retrospective cohort from National Women's Hospital, Auckland, in which women diagnosed with CIN3 between 1955 and 1976 were managed by biopsy alone or with limited treatment (a practice later judged unethical by the Cartwright Inquiry), the cumulative incidence of invasive cervical or vaginal cancers reached 31.3% at 30 years, rising to 50.3% among women with cytological evidence of persistent disease within 24 months of diagnosis [5]. A nationwide registry study of more than 10,000 women found that untreated CIN2 progressed to invasive cancer in 0.3% within 24 months and regressed to normal or CIN1 in 62.9% and progressed to CIN3 or persistent disease in 33.3% [6]. Despite the two studies not being directly equivalent, they establish that CIN3 carries a substantial lifetime risk of invasive cancer, whereas the majority of CIN2 regresses or remains non‐invasive, throughout a period where disease management decisions can be made. This difference justifies the immediate treatment of CIN3, while CIN2 can remain stable under active surveillance, particularly in younger patients, where harsh treatment is more consequential. These observations highlight the limitations of cytology and of nucleic acid amplification tests (NAATs), especially when used in isolation as stand‐alone tests. By combining tests, it is possible to overcome some of these limitations. Indeed, organized screening programs are credited with an 80% reduction in cervical cancer incidence in several countries [7, 8].

NAATs for cervical screening typically detect either viral DNA, which is abundant in the superficial layer of productive low‐grade squamous intraepithelial lesions (LSIL) where viral genome amplification and assembly are occurring, or viral transcripts (i.e. mRNA), such as those which encode the E6 and E7 proteins (Figure 1, 2). As the dual role of E6 and E7 as drivers of the productive life cycle and of oncogenic transformation is not always well appreciated, it is considered more fully below (see *Understanding HPV Disease Biology*). It is apparent however, that DNA and mRNA assays capture different aspects of the viral life cycle. DNA assays maximize sensitivity for hrHPV genome presence during its various replication and packaging stages, while mRNA assays selectively report E6/E7 transcriptional activity, which most likely underlies the modestly higher clinical specificity for HSIL seen in some studies [9]. These distinctions matter when choosing between DNA, mRNA or alternative approaches as primary screening tools, as well as when interpreting screening results during patient consultation. At present, WHO guidance identifies DNA testing as the default primary screening approach, which reflects its earlier introduction into the field, with mRNA testing now also recognized as a relevant alternative that has a distinct biological rationale for its use [10]. Triage tests typically focus on establishing disease severity or prognostic importance and often have a different rationale for their use.

**Figure 1:**
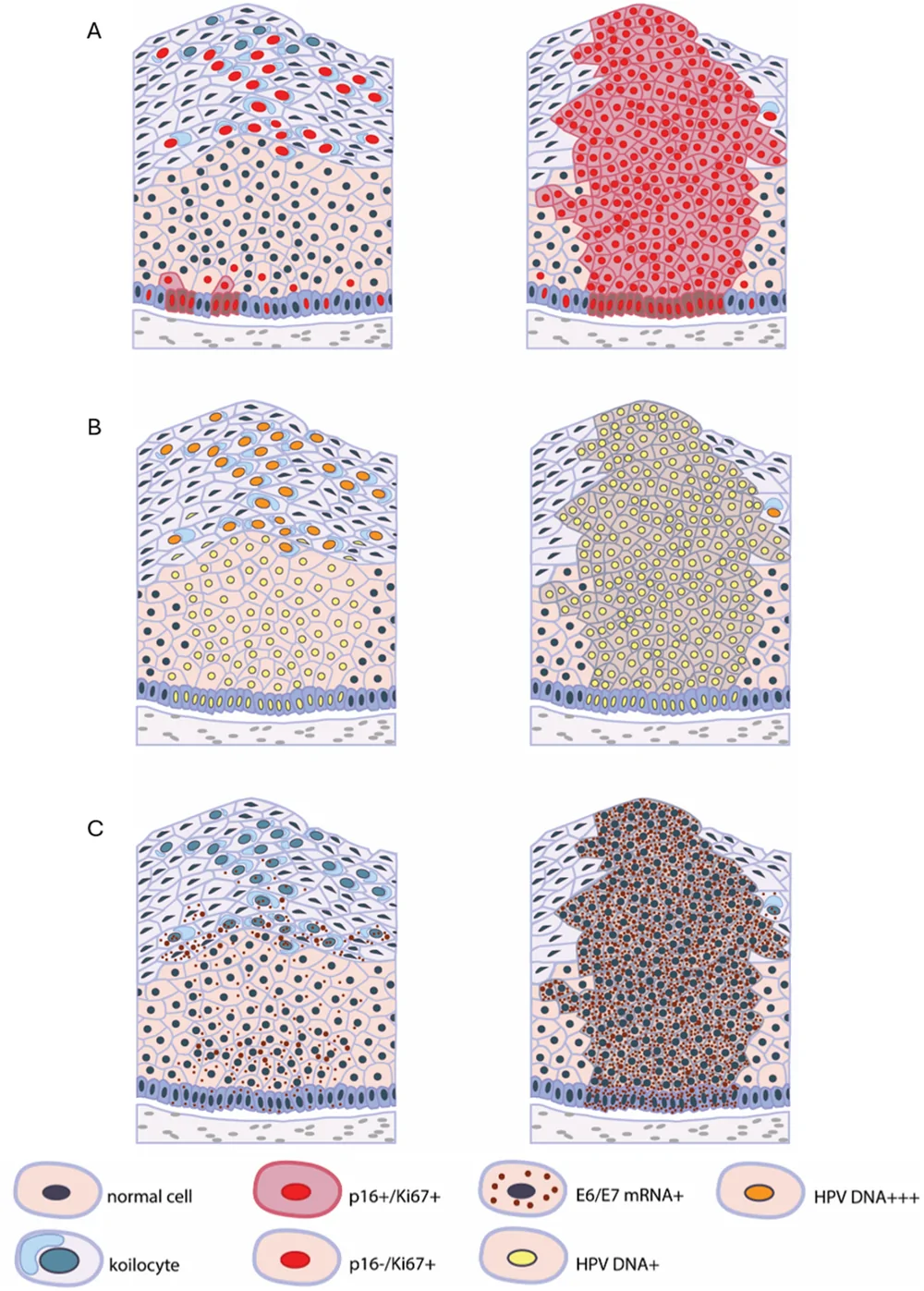
Spatial distribution of host and viral biomarkers in HPV‐infected epithelium of productive (LSIL) and transforming (HSIL) lesions. Schematic representation of marker distribution within the stratified squamous epithelium of LSILs (left) and HSILs (right), with the basement membrane at the base and the surface at the top. (A) Host p16/Ki‐67 co‐expression: p16 shown as red cytoplasmic signal, Ki‐67 as red nuclear signal. (B) Viral genomic DNA: cells undergoing productive genome amplification with high copy numbers shown as orange nuclei, cells in maintenance replication with low copy numbers shown as yellow nuclei. (C) Viral E6/E7 mRNA shown as brown punctate signal. The schematic depicts mature, stratified epithelium of the ectocervix and transformation zone. Although the expression patterns seen in immature metaplasia, the squamocolumnar junction and the cervical crypts are not depicted in this schematic, the concept of gene expression deregulation is explored in Figure 1, 2.

**Figure 2:**
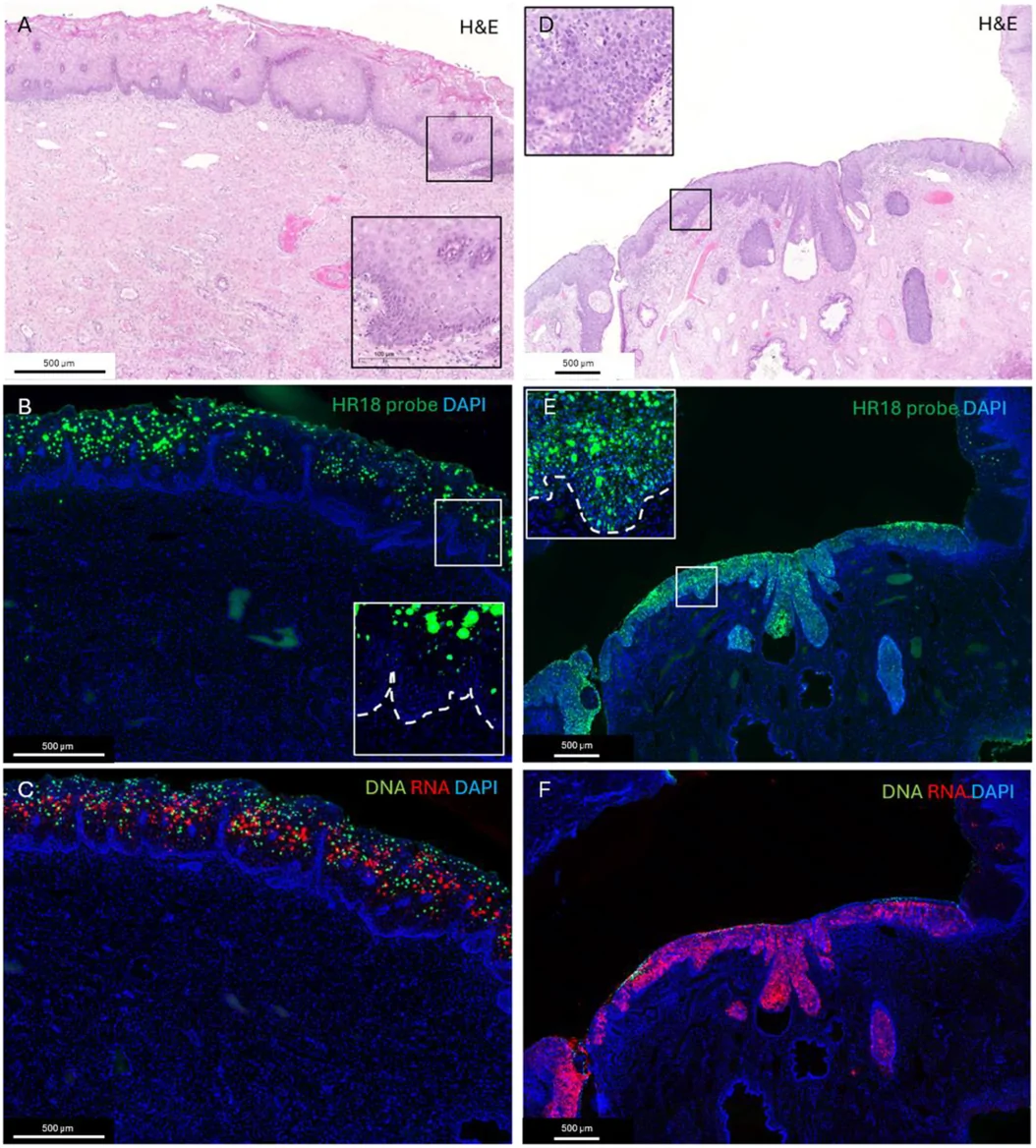
Spatial distribution of HPV genomes and E6/E7 transcription in LSIL and HSIL. Serial sections of LLETZ specimens showing low‐grade (LSIL; left column, A–C) and high‐grade (HSIL; right column, D–F) squamous intraepithelial lesions. (A, D) Haematoxylin and eosin staining. Scale bars, 500 µm. (B, E) HPV E6/E7 mRNA was detected by RNAscope® using the HR18 probe (ACD Bio, #312591). The DNA and RNA signals were separated by nuclease pre‐treatment of serial sections. (C, F) Pseudocolour composite overlays of E6/E7 mRNA (red) and viral DNA (green), generated by aligning channels from RNase‐treated (DNA) and DNase‐treated (mRNA) serial sections rather than from a single co‐stained section.

### 1.1 Understanding Human Papillomavirus Disease Biology in the Context of Screening Test Design and Test Performance

The viral E6 and E7 proteins serve a dual purpose that is central in understanding why DNA and mRNA assays perform differently across lesion grades. In productive lesions (LSIL), E6 and E7 drive differentiating suprabasal cells back into S‐phase, enabling viral genome amplification to take place. Their expression is spatially and temporally determined as infected basal cells divide and delaminate. In the basal compartment itself, the E6 and E7 proteins are required only at very low levels to modulate epithelial homeostasis and to maintain the reservoir of infection but must rise in abundance in the mid‐epithelial layers during genome amplification, before declining as vegetative replication gives way to genome packaging and the release of infectious particles from the epithelial surface. The presence of E6 and E7 is not in itself therefore a marker of oncogenic transformation [11], with both proteins expressed in productive (LSIL) and transforming (HSIL) infections. What differs is their spatial distribution. In transforming HSIL and CIN3, E6/E7 expression becomes deregulated in the basal layer, as well as throughout the suprabasal and more superficial epithelial layers, reflecting the prominent loss of differentiation‐dependent transcriptional control that defines such lesions [12].

Because the cervical cytobrush predominantly samples the superficial epithelial layers, the patterns of DNA and RNA it recovers are not equivalent [13, 14]. In a productive CIN1/LSIL for instance, viral DNA is found at high copy numbers in the mid‐ to upper epithelial layers, while viral E6/E7 mRNA transcripts are restricted to a band in the mid‐epithelial layers (Figures 1C and 2C). As a result, superficial cytobrush sampling will readily pick up viral DNA and infectious virions when sampling CIN1/LSIL, while E6/E7 mRNA transcripts will only be detected if cytobrush sampling extends into the mid epithelial layers. In transforming HSIL, viral DNA is present in the upper epithelial layers at low copy number, depending on whether genomes are episomal or integrated into the host cell chromosome at low copy number (e.g. as found in the SiHa cervical cancer cell line [15]), or as multi‐copy concatemers (as found in the CaSki cervical cancer cell line [15]), while E6/E7 mRNA is expressed at elevated levels throughout the full epithelial thickness [13, 14] (see Figures 1C and 2F). Transformation redistributes E6/E7 activity toward the epithelial surface while also reducing DNA copy number per‐cell when compared to LSIL, providing an explanation for the former's detection bias towards clinically relevant, rather than all, disease.

Figure 1, 2 illustrates the mature stratified squamous epithelium, as found on the ectocervix and in areas of mature squamous metaplasia within the transformation zone. Immature metaplasia and the regions around the squamocolumnar junction(s) have distinct patterns of gene expression deregulation that mark them apart from the productive LSIL. A cytobrush also samples the TZ and the endocervical canal, where the epithelium is not necessarily stratified and infected cells lie closer to the sampling surface. Transcripts could therefore be recovered from sites not represented here. However, lesions at the squamocolumnar junction and crypt entrances are more often high‐grade than productive and occur as thin HSILs [16] or HSILs at cervical crypt entrances [13], so mRNA recovered from these sites is more likely to reflect under‐recognized high‐grade disease than reduced test specificity. Instead, the magnitude of the specificity difference between DNA and mRNA assays can be explained by the population in which it was measured. In primary screening of the general population, where most women are hrHPV‐negative and correctly negative by both assays, specificity is high for both tests and the relative specificity of mRNA testing for CIN2+ is only ~1.03 [9]. In women referred with minor cytological abnormalities, nearly all are hrHPV‐positive and many have productive LSIL, the situation in which DNA and mRNA signals are predicted to diverge the most, and here mRNA testing was significantly more specific than HC2 [17].

An additional consideration when deciding between the type of nucleic acid test relates to the molecular stability of mRNA versus DNA, and the cellular origin of the sample being evaluated. HPV DNA packaged within viral capsids is protected from nuclease degradation by the structural L1/L2 proteins, so virions shed onto the cervical surface from adjacent productive infections can contribute detectable encapsidated DNA to a sample even where no local transforming infection exists [18]. E6/E7 mRNA, on the other hand, is not encapsidated and is detected only when transcriptionally active cells are sampled. A DNA‐positive, mRNA‐negative result may therefore reflect virion‐associated DNA from a transient or resolved infection, of low clinical significance, whereas an mRNA‐positive result, particularly in samples collected directly from the cervix, supports the presence of transcriptionally active infected cells in the sampled material. In higher lesion grades, vegetative viral genome amplification is largely absent because of the lack of cellular differentiation, and as a consequence, the average DNA copy number per cell is lower than in productive CIN1 [12]. The full‐thickness E6/E7 expression which is characteristic of HSIL can however be captured during cytobrush collection. The causal link between E6/E7 expression and disease severity underlies the modestly higher specificity of mRNA assays for CIN2+ (HSIL), while the abundant shedding of virion‐associated DNA in and around productive lesions underlie DNA test's marginal sensitivity advantage [19]. This distinction is particularly important for self‐collected specimens. Vaginal self‐samples or first‐void urine specimens do not contain material directly taken from the lesion, so the recovered material is shed material: encapsidated virions, cell‐free nucleic acid, and exfoliated cells. In this context, DNA detection is at an advantage over mRNA detection, as the latter requires a more preserved cellular integrity through collection and testing. With increasing use of self‐sampling, it is important to study the relative contributions of intact cells, cell‐free nucleic acid, and encapsidated virions in the signal detected.

The biological relationship between E6/E7 deregulation and cell cycle disruption in HSIL provides the mechanistic basis for the p16/Ki‐67 dual stain; a biomarker approach that can be used with liquid‐based cytology (LBC) samples, that acts as a surrogate marker of viral oncogene deregulation (Figures 1A and 3). p16 (encoded by the CDKN2A gene) is a cyclin‐dependent kinase inhibitor that normally maintains retinoblastoma protein (pRb) in its hypophosphorylated, growth‐suppressive state by binding to cyclin D. In the context of HPV infection, viral E7 binds and targets pRb for proteasomal degradation, releasing the E2F transcription factors necessary for cell cycle re‐entry. As a feedback response to pRb pathway‐disruption, p16 becomes overexpressed, allowing its use as a surrogate marker of E7 activity [20, 21]. It should be noted, however, that the mechanistic basis of E7‐driven cell cycle disruption is more complex than the classical pRb degradation model implies. Münger and colleagues have demonstrated that E7 retains oncogenic activity in pRb‐null cells through interaction with histone deacetylases and chromatin remodeling complexes to alter p16 levels and patterns of gene expression more generally through pRb‐independent pathways [22]. It appears that E7‐mediated transformation involves at least two parallel mechanisms which are regulated by pRb degradation and through direct chromatin remodeling. For the purpose of the dual stain however, p16 overexpression remains a relatively reliable surrogate for E7 activity regardless of which one of the downstream mechanisms predominates, since sustained functional loss of pRb drives p16 upregulation. Ki‐67 is a marker of active cellular proliferation, which is normally suppressed by the pRb pathway. In cells where E7 has degraded pRb, Ki‐67 expression persists even in differentiating suprabasal layers where it would normally be absent. Interestingly, the co‐expression of p16 and Ki‐67 within the same cell does not occur in normal keratinocyte biology, as p16 overexpression in an intact pRb pathway would suppress Ki‐67 expression and then proliferation. Their co‐detection in a single cell is a specific indicator for E7‐mediated disruption of the pRb‐p16‐E2F regulatory circuit, with p16/Ki‐67 expression generally regarded as an HPV‐specific marker of oncogenic transformation [20]. In the context of LBC, cells from productive LSIL (CIN1) show Ki‐67 positivity, with spatial restriction to the mid‐epithelial layers where E7 drives transient S‐phase re‐entry for viral genome amplification. These cells are largely below the surface accessible to cytobrush sampling, and do not co‐express p16 at levels indicative of sustained pRb pathway disruption [23]. In LBC samples from productive CIN1, surface‐derived cells are predominantly negative for both markers, whereas in HSIL (CIN2 + ) the two markers are increasingly co‐expressed in the upper epithelial layers accessible to cytobrush sampling (Figure 3). The dual stain thus interrogates host‐cell transformation, information that is biologically complementary to E6/E7 mRNA detection and more informative than viral DNA detection with regard to the assessment of disease severity. This complementarity may be particularly useful when mRNA testing is used as a primary screening option: mRNA positivity establishes that E6/E7 transcription is occurring, while dual‐stain positivity independently establishes that the downstream cellular consequences of sustained E7 activity are present, placing a woman at two mechanistically linked points along the transforming pathway [20, 24].

**Figure 3:**
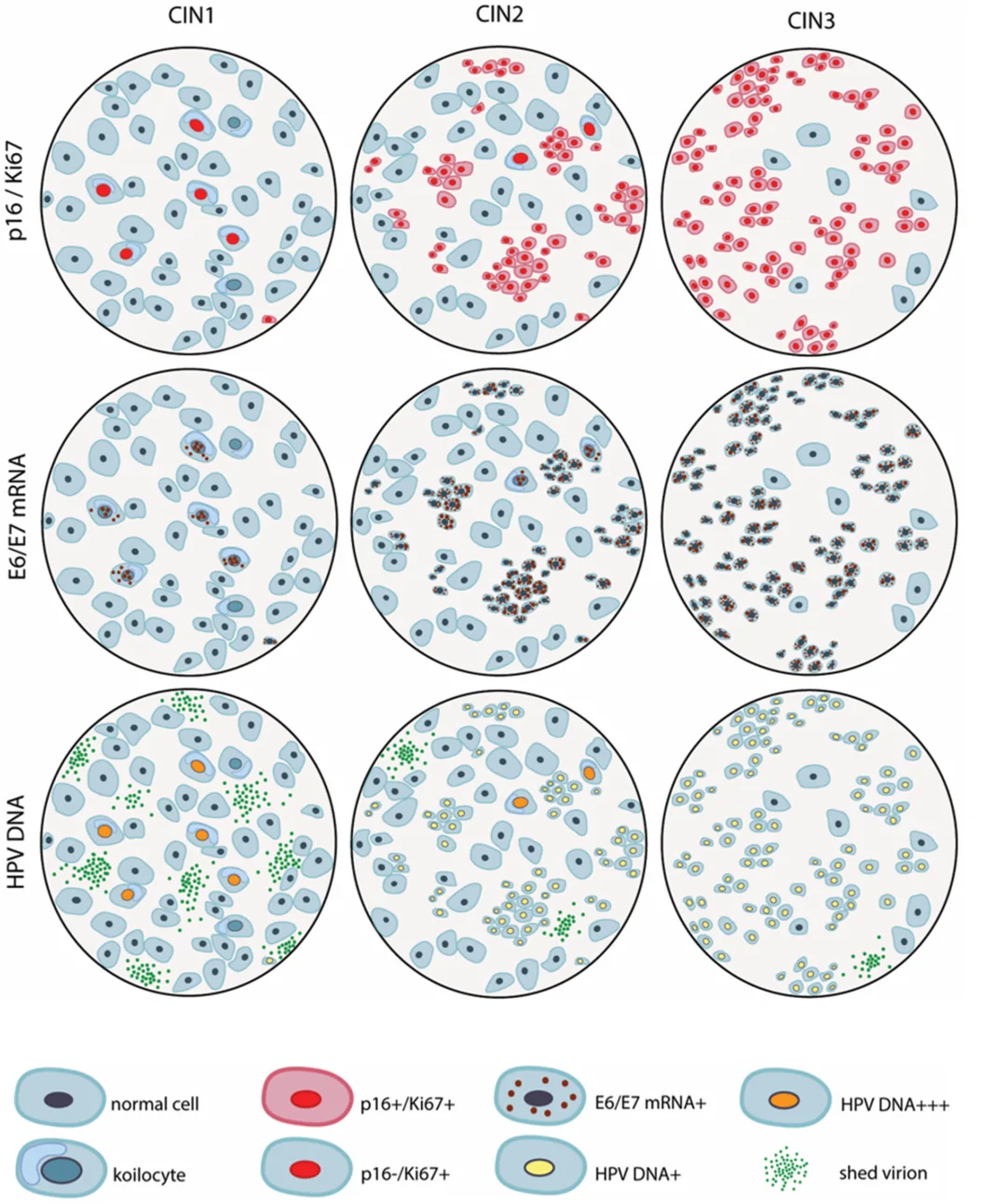
Schematic representation of the expected relative signals for HPV DNA, HPV E6/E7 mRNA, and p16/Ki‐67 dual‐stain cytology in liquid‐based cytology samples from CIN1, CIN2, and CIN3 lesions. The figure illustrates the relative detectability of each marker in cytobrush‐collected LBC samples. HPV DNA signals are strongest in CIN1, reflecting productive infection and high viral genome copy number in superficial epithelial cells. E6/E7 mRNA signals increase with lesion grade as viral oncogene expression becomes deregulated, while p16/Ki‐67 dual‐stain positivity rises from rare or absent in CIN1 to frequent in CIN2 and highest in CIN3, reflecting progressive host‐cell transformation. The figure represents expected relative signal taken from published data, not measured values.

## 2 Evolution of Nucleic Acid Tests and Their Utility in Routine Screening

### 2.1 Cytology and the Rationale for Molecular Screening

The goal of routine screening is to detect treatable pre‐cancerous lesions before they progress to invasive cancer [25]. Cervical cytology, building on the work of Papanicolaou, became the foundation of precancer detection, with LBC and digital imaging improving sample adequacy, workflow, and interpretation. Cytology is effective largely because CIN usually progresses slowly over many years, allowing repeated screening rounds to compensate for the moderate sensitivity of a single assessment, estimated at around 55–60% for conventional Pap smears depending on setting [26, 27]. NAATs, now the primary screening method in many programs, take a complementary approach: rather than grading disease severity, they detect the presence of HPV and thereby identify women at increased risk, particularly when infection is persistent. In current practice this is achieved using clinically validated, automated molecular platforms, such as the Roche Cobas system, Abbott Alinity m, BD Onclarity, Hologic Aptima, or, in point‐of‐care settings, Cepheid Xpert. These systems differ in workflow, throughput, specimen compatibility, and genotype reporting, but together they reflect the general shift of primary screening programs toward standardized, high‐throughput nucleic acid detection (Table S1); the assays listed are representative rather than exhaustive.

### 2.2 Persistence as the Determinant of Clinical Significance

Persistence, rather than a single positive result, is the key determinant of clinical significance. A single positive hrHPV test identifies viral presence but is not itself evidence of persistence, yet even a single positive result is associated with a significantly increased short‐term risk of subsequent high‐grade cytological abnormalities. In a study of more than 10,000 cytologically normal women followed over 2 years, HPV‐positive women at enrollment had an approximately 26‐fold increased risk of subsequently being found to have a high‐grade lesion as compared to HPV‐negative women. This rose to approximately 61‐fold for women who remained positive for any hrHPV type at follow‐up, and to over 800‐fold where the same type was detected at both timepoints (i.e. type‐specific persistence) [28]. These odds ratios describe risk of prevalent or incident high‐grade abnormality within a 2‐year window rather than lifetime cancer risk, but they illustrate how risk stratifies progressively with repeated detection and genotype‐specific persistence. In fact, several studies have now shown that repeated HPV testing is correspondingly more effective than cytology alone in reducing cervical cancer incidence and mortality [2, 19, 29], despite not directly identifying disease severity in the infected individual.

### 2.3 From Hybridization to Amplification

Detection of HPV genomes has long underpinned HPV diagnostics [30]. Early hybridization assays such as Southern blotting, *in situ* hybridization, and dot‐blot hybridization established the field but were poorly suited to routine use because of limited sensitivity, labor‐intensive workflows and low scalability [31, 32]. Nucleic acid amplification transformed diagnostics generally [33]: polymerase chain reaction (PCR) enables specific amplification of viral DNA and, when quantitative, allows viral genome copy number (often referred to as ‘virus load’) to be estimated, and detection thresholds to be tuned for clinical relevance. A recent study illustrates how this calibration is performed in practice, defining the type‐specific analytical thresholds: 3 IU/µl for HPV16/18, 25 IU/µl for HPV31/33/35/45/52/58 and 100 genome equivalents/µl for HPV39/51/56/59. These thresholds yielded 90.1% sensitivity, weighted for the type distribution in invasive cancer, at 92.0% cumulative specificity [34]. PCR and related methods, including transcription‐mediated amplification, nucleic acid sequence‐based amplification, and loop‐mediated isothermal amplification, now underpin many clinically validated commercial HPV assays (Table S1) [32]. Historically, the SPF10‐Line Probe Assay, and similar PCR‐based systems have been widely used in clinical trials and research, including the initial evaluation of vaccine efficacy [35]. A distinct landmark was the development of the Hybrid Capture 2 assay (HC2), which uses a signal amplification rather than a target amplification approach: RNA probes complementary to 13 hrHPV types hybridize to denatured viral DNA, and the resulting RNA:DNA hybrids are captured and detected by chemiluminescence without copying the target. HC2 served as the reference comparator in the large randomized European and North American screening trials [2, 36, 37, 38] and became an anchor test in the internationally recognized clinical validation framework [39], with prospective assays being required to demonstrate non‐inferior performance. The current situation is therefore best understood not as a choice between “HC2 and PCR”, but as a landscape of multiple validated assays performing broadly similar primary screening functions while differing in automation, genotype resolution, and intended use.

### 2.4 Clinical Validation and Regulatory Acceptance

Scientific validation and regulatory acceptance are separate hurdles, and only a subset of available assays is accepted for routine use in any given program. The internationally recognized benchmark remains the Meijer framework, which requires non‐inferior sensitivity and specificity for CIN2+ together with adequate reproducibility. Only a minority of commercially available HPV assays meet this standard in published studies, and national regulation then imposes an additional filter [25, 39, 40]. As a result, an assay may be analytically competent yet still not be accepted for routine screening in a particular country or for a particular intended use. Contemporary guidance recognizes both hrHPV DNA tests and E6/E7 mRNA tests as valid primary NAATs, with triage then used to identify women most likely to harbor clinically significant disease [9, 41].

These frameworks define clinical and not analytical validity. Clinically validated hrHPV assays are calibrated to detect infections that are most likely to be CIN2+ or CIN3+ with sensitivity of 90% while having enough specificity to limit colposcopy referrals. These tests are not designed to detect every single hrHPV infection, or every hrHPV type, and the non‐detection is a design feature, not a deficiency. Many assays fall short of the criteria because they are too analytically sensitive, detecting low copy‐number infections that have little clinical consequence, and losing specificity as a result. The analytical thresholds that optimize this trade‐off are described above and in [34]. Eleven hrHPV DNA assays meet the validation requirements, while only one mRNA assay has been fully validated [25], which is partly due to the criteria themselves being framed around DNA assays and imposing additional performance requirements on non‐DNA assays.

It is important to note a terminological implication following the viral biology described above. These assays detect presence of viral nucleic acid, not the presence of infection. DNA assays cannot distinguish between a transcriptionally active infected cell and encapsidated virions from adjacent resolved lesions. mRNA assays are better at distinguishing transcriptionally active cells, since transcripts are not encapsidated, but can also detect remaining transcripts in the cervix. It is an important distinction to make, as it defines the difference between viral presence and disease significance.

### 2.5 Epigenetic Biomarkers: Host and Viral Methylation

More recent additions to the landscape include self‐collected vaginal sampling, genotype‐informed risk stratification, and host‐cell DNA methylation assays such as QIAsure and GynTect. These methylation assays are targeted towards the detection of CIN2 + , and assessment of short‐term progression risk rather than HPV positivity alone [42, 43, 44]. The mechanistic basis lies in the ability of viral proteins to interfere with host DNA methyltransferase activity. HPV E7 binds DNMT1 directly and stimulates its methyltransferase activity, while sustained E6/E7 expression upregulates DNMT1 and DNMT3B [45, 46]. Hypermethylation accumulates at CpG islands of a number of host tumor‐suppressor promoters including CADM1 [47], MAL [48], and EPB41L3 [49], the tumor‐suppressive microRNA miR124‐2 [50], and FAM19A4 [51], the last identified through a genome‐wide methylation screen as a marker of HPV‐driven immortalization. Methylation levels of these targets are thought to be proportional to the time duration of transforming infection, with such modifications being largely absent in productive infections that have not yet begun to progress towards HSIL and cancer [46]. The HPV genome itself undergoes parallel methylation at CpG sites within the L1 and L2 late regions, increasing with disease severity and providing an independent molecular signal of the same transforming process [52]. Commercial assays now exploit host changes, viral changes, or both: host‐only panels such as QIAsure (FAM19A4/miR124‐2) and GynTect, alongside combined host‐plus‐viral classifiers such as S5, which scores methylation at EPB41L3 together with L1/L2 regions of HPV16, 18, 31 and 33 [53]. The clinical role of methylation is likely to extend beyond triage: in CIN2 lesions under active surveillance, methylation‐negative women have a high probability of spontaneous regression, whereas methylation‐positive women carry substantially elevated progression risk [54], supporting a future role in distinguishing lesions for treatment from those for surveillance. Methylation assays also perform comparably on self‐collected vaginal samples and first‐void urine, positioning them as a natural triage partner for the self‐sampling expansion now reshaping cervical screening programmes [55, 56].

## 3 Comparative Strengths and Limitations of Current Screening and Triage Methods

Each approach for HPV screening has distinct characteristics that affect its role in clinical performance. DNA‐based NAATs, including PCR and the HC2 assay, offer high diagnostic sensitivity across a broad range of hrHPV types and detect viral presence that cytology alone would miss [19, 57]. Their principal limitation is lower specificity for clinically relevant disease: because they detect transient as well as persistent infection, they can lead to over‐referral for colposcopy and a risk of unnecessary intervention where no triage strategy is applied [58]. This has important practical and clinical consequences, including increased pressure on colposcopy services and the possibility that additional diagnostic biopsies may be collected unnecessarily. Such follow‐up procedures in women who ultimately have little or no histological disease are not ideal, and can lead to avoidable psychological distress, including anxiety and concerns regarding fertility [10, 59, 60]. Genotyping partly mitigates these problems, as each HPV type has a different risk of persistence and carcinogenic potential, supporting risk‐based partitioning rather than treating all hrHPV infections as equivalent [13, 61]. DNA based assays are also particularly useful when direct sampling of the cervix is not possible or difficult. The presence of viral DNA and encapsidated genomes collected from nearby sites may be used as an “indirect” indicator of cervical infection, and in the setting of self‐sampling, this convenience and acceptability can outweigh the greater precision of direct cervical sampling [62, 63].

For clinician‐collected cervical samples, validated mRNA assays can offer enhanced specificity by preferentially distinguishing transcriptionally active infections rather than infections of lower clinical relevance [64]. This discriminatory power follows directly from current understanding of HPV biology: E6/E7 transcriptional activity is more tightly linked to oncogenic progression than genome presence, so DNA‐positive, mRNA‐negative women represent a lower‐risk group than double positive women [65, 66]. mRNA testing can therefore be used either in primary screening as an alternative to DNA, or as a triage tool for women with abnormal cytology [9, 67]. Practical limitations remain and are operationally significant. The Aptima assay for instance, was originally validated for samples collected in ThinPrep PreservCyt medium, and although a validated SurePath protocol has since been approved within the NHS Cervical Screening Program, this imposes considerably shorter specimen transfer windows. Other sample collection and transportation requirements, and the absence of standardized cellular adequacy controls have limited applicability in some settings, highlighting why clinical adoption of mRNA testing has been slower and more geographically uneven than its performance data alone would predict [68].

Cytology, including Pap smears and LBC, provides a morphological assessment of exfoliated cells and is more targeted to establishment of disease status than DNA testing, because it directly identifies the cellular changes that define neoplastic progression (Figure 3) [69]. However, cytology has traditionally been limited by inter‐observer variability, sampling inconsistency, and the subjectivity of the test. Recent advances in digital pathology have however enabled the development of AI‐assisted cytological screening platforms, (e.g. Genius Digital Diagnostics System, Hologic; https://www.hologic.com/hologic-products/cytology/genius-digital-diagnostics-system), that combine automated volumetric imaging of liquid‐based cytology preparations with deep learning algorithms trained to identify and prioritize abnormal cells for review. These systems have been reported to improve sensitivity and throughput relative to manual review while increasing result consistency [70, 71]. However, AI‐assisted systems require rigorous analytical and clinical validation before widespread adoption, and expert oversight remains essential given the current regulatory and quality assurance frameworks around cervical screening.

Overall, no single test provides an optimal balance of sensitivity, specificity, and feasibility, and the direction of routine practice is therefore toward better risk stratification after a positive HPV result, using a combination of HPV detection, genotyping, cytology and biomarker staining [10]. Several validated combinations that reflect HPV disease biology are already in use. Primary hrHPV testing followed by immediate colposcopy for HPV16/18‐positive women, with cytology triage for other hrHPV types, combines the strengths and limitations of each approach into a workable scheme, particularly for women aged 30 and older in whom a positive result is more likely to represent persistent, clinically relevant infection [2, 24]. Primary hrHPV testing followed by p16/Ki‐67 dual‐stain triage (e.g. CINtec PLUS Cytology Kit, Roche, https://diagnostics.roche.com/global/en/products/lab/cintec-plus-cytology-us-rtd000761.html) introduces a biomarker‐based follow‐up that is conceptually close to the direct detection of HPV transcripts, and approaches that combine extended genotyping with dual‐stain cytology extend this logic further [72]. The unifying principle is that the most informative combinations interrogate different layers of risk, viral presence, viral oncogenic activity, and host‐cell transformation, although the definitive confirmation of disease still ultimately requires cytological or histological assessment [73, 74].

## 4 Heterogeneity of HPV‐Associated Disease and Clinical Interpretation of HPV Nucleic Acid Tests

### 4.1 Intra‐ and Inter‐Lesional Heterogeneity and the Loss of Spatial Information in Bulk Assays

Biological heterogeneity amongst the cervical lesions within an individual is a significant limitation when interpretating nucleic acid test results. Disease severity, infecting HPV type, integration status and patterns of viral gene expression can vary not only between patients but also at different cervical locations in the same woman. Areas of productive LSIL, partially transforming epithelium, and fully transforming HSIL/CIN3 can coexist within millimeters of one another, and may reflect either the sequential stages of a single infection or two biologically distinct infections with different hrHPV types [75]. This intra‐ and inter‐lesional variability challenges the sensitivity and specificity of molecular diagnostic techniques, particularly when the biological question extends beyond the simple presence or absence of hrHPV.

Traditional “grind and bind” bulk NAAT assays, whether DNA‐ or RNA‐based, provide an average signal from the homogenized specimen. The familiar analogy is a smoothie made from a mixed fruit bowl: the blended product contains the same constituents as the original bowl, and their total quantities are preserved, while the information about quantities of which fruit it came from, how ripe each individual piece was, and the spatial arrangement of the original components is irreversibly lost (Figure 4). For primary screening, where the question is whether a clinically relevant hrHPV infection is present, this averaging is an acceptable trade‐off given the need for high throughput and a low per person cost. However, for triage, where the question shifts from the virus presence to biological significance, grade of neoplasia and prognosis, the loss of spatial and cellular resolution is far more consequential. The granular information preserved in cytological samples, which facilitates morphological assessment and the identification and grading of even small numbers of abnormal cells, is degraded when the original sample is dissolved and processed for ‘bulk’ nucleic acid extraction. A small focus of CIN3/HSIL amongst a more extensive region of low‐grade neoplasia, is likely to be masked at the ‘overall’ mRNA or methylation detection level, by the dominant LSIL signal. As reported, the apparent “progression” from CIN1 to CIN3 on follow‐up can reflect the initial presence of an undetected synchronous CIN3 focus rather than true biological progression [75]. This problem is amplified where a positive NAAT is used as the sole basis for excision, as in some screen‐and‐treat settings, because the result carries no information on whether the underlying lesion is productive and likely to regress or transforming and in need of intervention [11, 76]. The 2021 WHO guideline acknowledges this problem by suggesting screen‐and‐treat and screen‐triage‐and‐treat as equally acceptable strategies for the general population, but suggests specifically the screen‐triage‐and‐treat approach for women living with HIV – where the higher prevalence of mixed‐grade and persistent lesions makes the consequences of information loss from bulk averaging most acute – on the basis that triage reduces unnecessary treatment and the associated risk of preterm delivery, without meaningful loss of cancer prevention effectiveness [10].

**Figure 4:**
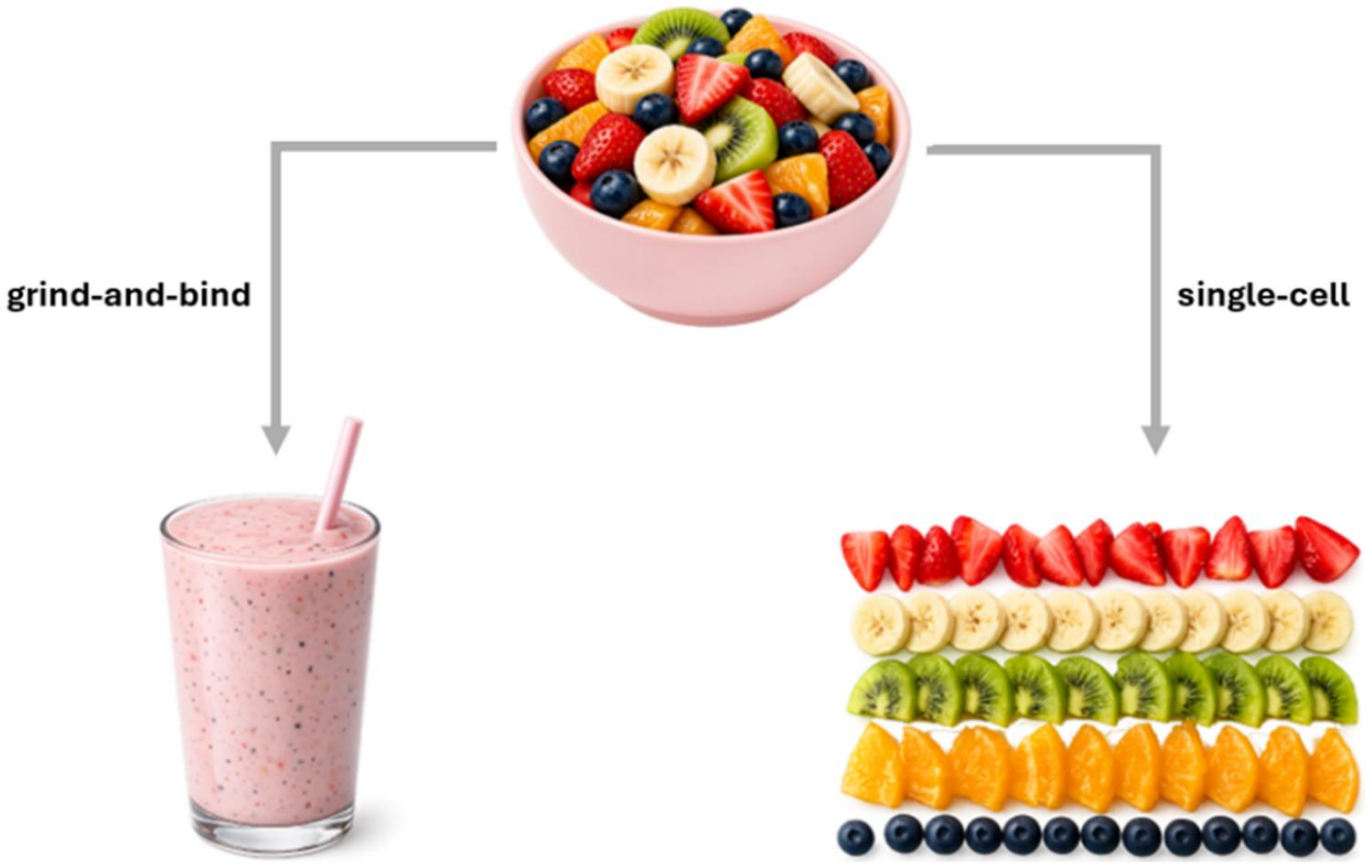
Conceptual illustration of bulk “grind‐and‐bind” versus single‐cell analysis. The mixed fruit bowl represents a biologically heterogeneous specimen. Homogenization into a smoothie symbolizes conventional bulk molecular testing, in which all cellular material is combined and measured as an average signal. By contrast, separation of the fruit into individual components symbolizes single‐cell approaches, which preserve heterogeneity and enable detection of distinct and potentially clinically important subpopulations that may be masked in bulk assays.

### 4.2 Sampling Variation and Interpretive Reproducibility

Sampling can lead to another dimension of variability that directly affects the risk estimates on which disease management depends. A cytobrush exfoliates cells from the cell layers at the surface of the cervix which might contain lesions of several different grades, while a colposcopy samples only a fraction of that area. This can make the interpretation of the results difficult. In the ASCUS‐LSIL Triage Study (ALTS), it was found that well‐trained pathologists only moderately agree on cytological and histological interpretation [77], identifying a problem with reproducibility. The 2‐year cumulative risk of CIN2/3 following an LSIL was 27.6% [78], showing that a low‐grade result identified through cytology does not reliably indicate the presence of lesions that are productive and will regress. Discordance between colposcopic assessment and excision occurs in both directions. Punch biopsy can underestimate lesion grade: in the Gardasil trials, CIN3 or AIS on excision was under‐called in around 40% of cases [79]. Conversely, a large proportion of excisions performed for suspected or biopsy‐confirmed HSIL contain no high‐grade disease. In a series of 559 women treated by LEEP for CIN2 or CIN3 on punch biopsy, no lesion was present in the excised specimen in 18.2% of cases, and this was more likely where the biopsy showed CIN2, and where types other than HPV16 were present [80]. These findings are consistent with colposcopic over‐calling, removal of small lesions by the biopsy itself, and regression between biopsy and treatment. Nucleic acid test results are thus assessed against a reference standard that is not ideal, which may be reflected in the small performance variations of the different assay types.

### 4.3 Heterogeneity in Immunocompromised Populations

Mixed‐grade heterogeneity is especially common in immunocompromised populations. In HIV‐positive women not on suppressive antiretroviral therapy, impaired immune surveillance leads to higher HPV persistence, more frequent infection with multiple genotypes, and more complex lesional landscapes in which productive and transforming areas coexist [81, 82], a situation that is not fully resolved even after antiretroviral drugs are administered. HIV‐positive women show higher HPV acquisition and lower clearance than HIV‐negative women, with acquisition associated with reduced local CD4 T‐cell counts and a higher incidence of both LSIL and HSIL [83]. In general, HPV infections are first detected following the initial exposure to hrHPV types as a young adult; a population which is immunologically naïve to high‐risk HPV and the antigens that they express [11]. As initial immune ignorance transitions through immune detection and eventually to immune control, most LSIL and much of the HSIL that arises soon after infection is brought under control [84], in line with the observation that disease typically clears in this age group by 18 months post infection [85], even though HPV DNA may persist subclinically [11]. In this situation, a repeat positive is detecting viral DNA, which is not the same as a continuing productive or transforming infection. Encapsidated genomes and residual nucleic acid can sustain a positive result in the absence of active disease, which has led to the adoption of ‘validated tests’ with an ‘adjusted sensitivity’ designed to ensure the detection of most clinically relevant disease, which restrict the number of false positives rather than maximizing analytical sensitivity [25, 39]. In the presence of E6/E7 transcripts, ongoing infection with transcriptionally active cells is considerably more likely. This difference between the two analytes justifies in part why repeat testing with a single assay type might not resolve the question the clinician is actually trying to answer and is a part of the rationale for combining different assays. In HIV‐infected women, this process takes longer or may not occur effectively. From these observations, it is clear that the age‐related natural history of infection, the timing and the extent of post‐infection immune control, and variability in an individual's exposure to new HPV types, will all influence whether an individual tests positive at different points in their life. The realization that papillomavirus infections can remain subclinical with periodic fluctuations in detectability impact significantly on how HPV testing is interpreted. Although the utility of HPV detection is generally considered at a population level, with broadly based follow up algorithms [10, 24], the need to consider results and treatment options at an individual level is persuasive, and if resources allow, clinical consultation and treatment options should be aligned with individual patient history and patient choice.

### 4.4 Recovering Spatial Information: In Situ Approaches

Recovering the spatial information lost to bulk assays is increasingly feasible with in situ technologies. RNA *in situ* hybridization methods such as RNAscope, together with fluorescent *in situ* sequencing and related spatial‐transcriptomic approaches, preserve tissue architecture while localizing viral nucleic acids to individual cells [86, 87]. Applied to formalin‐fixed cervical sections, RNAscope has shown that E6/E7 mRNA and viral DNA are not uniformly distributed within the same cells in productive CIN1, has confirmed the redistribution of E6/E7 expression that characterizes transforming HSIL, and has identified site‐specific differences in viral pathology at cervical crypt entrances that may explain their disproportionate association with high‐grade disease [13, 14]. The method uses paired oligonucleotide probes whose adjacent binding is required to trigger signal amplification, which suppresses background and allows single transcripts to be visualized, although reliable performance requires careful optimization for each tissue and fixation protocol. These approaches are research methodologies rather than validated screening or triage tests; their value is, therefore, mechanistic rather than diagnostic. When applied to cervical tissue (Figure 1, 2), these approaches map E6/E7 mRNA and HPV DNA to individual epithelial cells and correlate them with morphology and lesion grade, providing a level of resolution inaccessible to bulk NAATs and a direct experimental confirmation of the compartmentalization that this review argues governs assay performance.

## 5 Refining Screening and Triage Systems: CCL, Spatial Methodologies and Triage Innovations

Ideally, future developments in cervical screening should make better use of the spatial and cellular information that bulk NAATs discard. The move towards single‐cell and/or spatial approaches has already impacted research and is being driven to a large extent by advances in protein and RNA detection methods, and in imaging technology. Spatial methods retain tissue integrity so that interactions between cell populations can be interpreted, but for routine use, time and cost implications restrict implementation. As a triage tool, the AI‐assisted evaluation of LBC images is a step in the right direction however, since it links HPV detection to cell morphology and lesion severity without specialized spatial assays. Additional biomarkers that may be proposed for routine use, must be available in a clinically validated format with continuity of reagent supply [9]. The p16/Ki‐67 dual stain meets this requirement and has been applied successfully to cervical pathology and to LBC, recently in combination with digital imaging that grades the extent and number of positive cells [88]. Its limitations are however instructive: p16/Ki‐67 positivity is not confined to HSIL and may also occur in LSIL, while surface‐proximal cells that are p16‐negative may still carry disease‐relevant morphological information, such as koilocytosis, mitotic figures and an increased nuclear‐to‐cytoplasmic ratio. This again points to the need for multiple complementary markers, balanced against the cost and time constraints that govern routine implementation.

A promising route is to extend spatial mapping to the cervical surface itself, where underlying disease can be inferred from the pattern of biomarkers and the morphology of exfoliating cells [89]. The virally encoded E4 and L1 proteins are found at the surface of low‐grade productive infection, whereas in transforming HSIL these are replaced by p16/Ki‐67 double‐positive cells; combining biomarker and morphological information allows these states to be distinguished. The cervical cell‐lift (CCL) approach uses an adhesive transparent membrane that, pressed onto the cervix and when peeled away, lifts off a sheet of cells without disrupting their spatial organization. Biomarker and/or morphologically defined abnormal cells can be assessed according to their proximity to each other, with HPV presence and HPV type being established independently using the same sample. Depending on resources, the analysis can be as simple as a Pap stain visualized as an adjunct to VIA or can be extended to include spatial and transcriptomic analysis if required. Fine cytobrush sampling of the endocervical canal can be used additionally to target the less accessible part of the cervical transformation zone, and the crypt entrances, where site‐specific differences in viral pathology favor the development of high‐grade disease [13, 90]. Indeed, the capacity to resolve transcriptional heterogeneity at cellular and sub‐regional resolution is most valuable at the cervical transformation zone, where site‐specific differences in viral pathology, along with microenvironmental cues, are thought to influence the site's vulnerability to HPV‐induced neoplasia [13].

The requirement for a separate endocervical sample is a limitation of CCL. The membrane removes a cell layer from the ectocervical surface but cannot reach the inside of the canal or into the cervical crypts. So, lesions arising above the SCJ are not detected, which especially affects women in whom the TZ is inside the canal, such as older and post‐menopausal women, and women who have undergone prior excisional treatments. Fine endocervical brushes are already used routinely in many screening programs and can be combined with CCL, allowing the ectocervical spatial map and the endocervical sample to be interpreted together. The disadvantages of this are additional costs and discomfort associated with the endocervical brush. As CCL is proposed as a triage method rather than primary screening however, the requirement for an additional sampling device is less limiting than in the screening stage.

A coordinated combination of bulk “grind‐and‐bind” screening with targeted spatial analysis, informed where possible by local immune status and methylation as an indicator of past E6/E7 deregulation, is therefore the most plausible near‐term direction. Implementation will depend on local resources and cost–benefit considerations, and in the future, on the population‐level changes in HPV prevalence and precancer incidence following the introduction of prophylactic vaccination.

### 5.1 Screening in a Setting of Declining Prevalence

With the prevalence of cervical disease now changing rapidly in countries where vaccination has been implemented [91], screening performance and approaches are being reconsidered [92]. The predictive value of tests falls as prevalence falls, even with unchanged sensitivity and specificity [93]. Cytology, for example, loses positive predictive value as high‐grade disease becomes rarer, and the positive predictive value of abnormal cytology for CIN2+ has already fallen in vaccinated cohorts in Australia and Sweden [94, 95]. Declining prevalence may also reduce its sensitivity, as abnormalities become too rare for reliable detection by cytology readers [96]. Primary hrHPV testing is likely to follow a similar trend [97].

In England, cohorts vaccinated with the bivalent vaccine at 12‐13 years have shown reductions of around 87% in cervical cancer and 97% in CIN3 compared with unvaccinated cohorts [98]. CIN3 has thus already fallen by more than an order of magnitude. In Scotland, no invasive cancers were identified up to July 2020 among women born 1988‐1996 who were vaccinated at 12–13 years [99]. hrHPV positivity falls less because non‐vaccine types continue to circulate, and the residual HPV‐positive population is enriched for less carcinogenic types [92]. Although a discussion of screening algorithms and how they may change is somewhat beyond the scope of this review [92, 100], it is worth considering which aspects of viral biology matter most. Genotyping remains important because vaccination does not alter the risk posed by an existing infection [92]. However, as HPV16/18 become rare, a growing proportion of HPV‐positive women carry lower‐risk types whose infections are less likely to progress, and bulk assays cannot distinguish productive from transforming infection. Markers of viral oncogenic activity and host‐cell transformation are therefore expected to become more informative [92]. Methods that recover spatial and cellular information, although costly at present, are in principle well suited to this, particularly when applied to a shrinking triage population.

In summary, the differing performance of HPV DNA‐ and RNA‐based assays is best understood as a consequence of the spatial organization of events during productive and transforming infection within the cervical epithelium. DNA assays maximize sensitivity for viral presence, whereas mRNA assays provide greater specificity for transcriptionally active disease, with neither alone fully capturing disease significance. The most effective screening pathways therefore combine primary HPV detection with complementary triage methods that interrogate genotype, host‐cell transformation, and morphology. The performance ceiling of any cervical screening assay is set by the spatial and differentiation‐dependent compartmentalization of the HPV life cycle. Future gains will come from methods that recover, rather than average over, that biological detail.

## Ethics Statement

Ethical approval for the use of clinical cervical lesion specimens was granted by the Human Research Ethics Committee (Medical), Faculty of Health Sciences, University of the Witwatersrand, Johannesburg, South Africa. The specimens were transferred to the University of Cambridge under a material transfer agreement for human biological materials executed on 27 October 2020. Samples were de‐identified before transfer and were received, stored and processed in accordance with the Human Tissue Act 2004, applicable Human Tissue Authority requirements and University of Cambridge institutional governance procedures [under HTA licence number 12196]. The cervical lesion cohort used for the analyses presented in Figure 1, 2 has been described in detail previously [90].

## Supporting information


Supporting File

## Data Availability

The data that support the findings of this study are available from the corresponding author upon reasonable request.
