# Supplementary material for: Productive or Transforming? How Epithelial Compartmentalization of HPV DNA and E6/E7 Transcripts Shapes Cervical Screening Assay Performance
Source: J Med Virol. 2026 Oct 27;98(11):e71196. doi: 10.1002/jmv.71196 (PMC13711476; doi:10.1002/jmv.71196)
Supplement: Supplementary file 1 [file JMV-98-e71196-s001.docx]

## Supplementary Table

Table 1. Overview of nucleic acid-based and complementary HPV testing approaches currently available for cervical cancer screening and triage.

| **Test type** | **Commercial examples** | **Mode of function** | **Analyte detected and its biological origin** | **Typical use** |
| --- | --- | --- | --- | --- |
| **DNA** signal amplification / hybrid capture (pooled hrHPV) | Qiagen Digene HC2, Qiagen careHPV | Nucleic acid hybridisation with signal amplification; detects pooled hrHPV DNA rather than genotype-level | Pooled hrHPV genomic DNA without target amplification. Recovers both encapsidated virion DNA shed from productive lesions and intracellular episomal or integrated DNA from transforming infection; does not distinguish life-cycle stage. | Historical and still used in **primary screening**. careHPV was designed for lower resource settings |
| **DNA** PCR, pooled hrHPV with partial genotyping | Roche cobas HPV, Abbott RealTime HR HPV | PCR-based detection of hrHPV DNA; usually reports HPV16 and HPV18, with the remaining types pooled | hrHPV genomic DNA with HPV16 and HPV18 reported separately. Same biological analyte as HC2: encapsidated and intracellular viral DNA across productive and transforming lesions; type-specific reporting reflects oncogenic potential, not life-cycle stage. | **Primary screening**; mainstream |
| **DNA** PCR with extended genotyping | Onclarity, Abbott Alinity m HR HPV, Cepheid Xpert HPV | PCR-based detection of hrHPV DNA; Onclarity reports 6 individual types plus 3 grouped channels; Alinity m reports HPV16, 18 and 45 individually plus 2 grouped channels; Xpert reports HPV16 and HPV18/45 plus 3 grouped channels | hrHPV genomic DNA with individual reporting of additional high-risk types. As above; extended genotyping refines type-specific persistence risk but does not differentiate productive from transforming infection. | **Primary screening** with risk stratification |
| **mRNA** E6/E7 detection with broad genotype panel | Hologic Aptima HPV Assay | Detects E6/E7 mRNA, aiming to identify transcriptionally active infections linked to transformation | E6/E7 oncoprotein mRNA from 14 hrHPV types. In productive infection, transcription is confined to a mid-epithelial band partially below cytobrush sampling depth; deregulated full-thickness E6/E7 expression in transforming HSIL/CIN3 reaches the surface and is sampled. | **Primary screening** in some settings; **triage** in cytology-based screening programmes |
| **mRNA** E6/E7 detection with limited genotype panel | PreTect HPV-Proofer | Detects E6/E7 mRNA from a narrower set of hrHPV types | E6/E7 mRNA from a smaller hrHPV type set. Same biological signal as Aptima but reduced genotype coverage; transcriptional activity again concentrated in the mid-epithelial layers of productive infection and redistributed to the full epithelial thickness in transforming infection. | **Triage-oriented**; less popular than Aptima |
| **Self-collected** HPV testing, PCR-based **DNA** test | cobas HPV and BD Onclarity | Vaginal self-sample collected by the patient, then tested by a validated HPV assay, most often a PCR-based DNA test | hrHPV genomic DNA recovered from the vaginal milieu. Signal is thought to derive largely from encapsidated virions shed from cervical infection, whose L1/L2 capsid protects viral DNA against nucleases and enables detection at a site distant from the lesion. | Expanded access for under-screened groups; **organised screening** and outreach |
| **mRNA** reflex genotyping tests after a positive screen | Aptima HPV16 18/45 Genotype Assay | Reflex genotyping after a positive broad HPV result | E6/E7 mRNA from HPV16, 18 and 45, with HPV16 reported separately and HPV18 and 45 reported together. Detects transcriptionally active infection by the highest-risk genotypes most strongly associated with progression to invasive disease. | **Triage** and management of HPV-positive women |
| **DNA methylation** | QIAsure (FAM19A4 / miR124-2), GynTect, S5 classifier | Detects host methylation changes associated with persistent transforming infection rather than HPV presence itself | Host CpG methylation at HPV-driven tumour-suppressor promoters (e.g. FAM19A4, miR124-2, EPB41L3, CADM1, MAL); S5 additionally scores methylation of HPV16/18/31/33 L1/L2 regions. Downstream consequence of sustained E6/E7 deregulation acting via E7–DNMT1 interaction; accumulates with the depth and duration of transforming infection. | **Triage** and management of HPV-positive women |
| **Biomarker detection** | CINtec PLUS Cytology | Detects p16/Ki-67 dual staining in the same cell, indicating transforming infection | Host p16 and Ki-67 co-expression in a single cell. Surrogate for E7-mediated pRb degradation and the consequent unrestrained suprabasal proliferation that characterises transforming infection; co-expression does not occur in normal keratinocyte biology. | **Triage** and management of HPV-positive women |
| **First-void urine** hrHPV DNA testing | Colli-Pee collection device in combination with Abbott RealTime | The first fraction of urine is collected which is then tested with an HPV DNA-based assay | hrHPV genomic DNA recovered from the first urine fraction. Thought to derive largely from encapsidated virions and exfoliated cells shed from the cervix and vagina and washed out in the initial void; their relative contributions have not been established; capsid stabilisation supports detection at a sampling site remote from the lesion. | **Emerging self-sampling** approach for people who do not want speculum-based screening; **organised screening** and outreach |
| **Investigational** | | | | |
| **Self-collected** HPV testing, mRNA test | Hologic Aptima HPV Assay on vaginal self-samples | Vaginal self-sample collected by the patient and tested for E6/E7 mRNA; performance depends on the collection device and pre-analytical handling of the sample | E6/E7 mRNA from 14 hrHPV types in exfoliated cells. Transcripts require intact, transcriptionally active cells, which are sparse in a sample taken away from a lesion; this probably underlies the lower sensitivity for CIN2+ than on clinician-collected samples. | **Investigational**  not yet validated for routine self-sampling |
| **First-void urine** mRNA testing | Hologic Aptima HPV Assay on first-void urine | First-void urine collected by the patient (e.g. with the Colli-Pee device) and tested for E6/E7 mRNA, in some studies after pre-treatment to improve RNA recovery | E6/E7 mRNA from exfoliated cells in first-void urine. Urine contains few intact cervical cells, and unprotected RNA degrades without an appropriate preservative, so the signal is weaker and less consistent than the DNA equivalent. | **Investigational**  not currently suitable for screening due to low sensitivity |

## Supplementary Methods

RNAscope and Nuclease Treatment

RNAscope was performed using reagents and probes from Advanced Cell Diagnostics (ACD) following the manufacturer's protocol for the RNAscope 2.5 HD Brown Single Plex Kit, with the modifications described below. A single probe set, RNAscope HPV-HR18 (ACD; #312591), was used to detect both HPV DNA and E6/E7 mRNA. Serial FFPE sections [5 µm] were baked overnight at 60°C, deparaffinised in fresh xylene (2 × 5 min), and transferred to 100% ethanol (2 × 1 min) before air-drying. Endogenous peroxidase was blocked using the kit-provided H₂O₂ reagent (10 min, 40°C). Target retrieval was performed in RNAscope® Target Retrieval Reagent in a decloaking chamber (15 min, 100°C); slides were then briefly rinsed, placed in 100% ethanol (3 min, room temperature), and air-dried. A hydrophobic barrier was applied.

Protease treatment was performed with RNAscope® Protease Plus (10 min, 40°C). To distinguish the two nucleic acid species, adjacent serial sections were then treated with nuclease before hybridisation. To detect mRNA, DNA was removed with DNase I (Thermo Fisher Scientific; EN0521; 30 min, 37°C). To detect viral DNA, RNA was removed with RNase A (Qiagen; 1006657) and RNase T1 (Fermentas; EN0542) (30 min, 37°C). Probe hybridisation and amplification steps were performed according to the ACD manual. For Figure 2, images of DNase- and RNase-treated serial sections were aligned in Adobe Photoshop and assigned pseudocolours (mRNA, red; DNA, green) to generate the composite overlays. This method is validated in [1].

1.	Pronin, A., et al., *Expression of olfactory signaling genes in the eye.* PloS one, 2014. **9**(4): p. e96435.
